# Effects of endurance training on skeletal muscle mitochondrial function in Huntington disease patients

**DOI:** 10.1186/s13023-017-0740-z

**Published:** 2017-12-19

**Authors:** Sandro Manuel Mueller, Saskia Maria Gehrig, Jens A. Petersen, Sebastian Frese, Violeta Mihaylova, Maria Ligon-Auer, Natalia Khmara, Jean-Marc Nuoffer, André Schaller, Carsten Lundby, Marco Toigo, Hans H. Jung

**Affiliations:** 1 Department of Neurology, University Hospital Zurich, , University of Zurich, Frauenklinikstrasse 26, 8091 Zurich, Switzerland; 2 Institute of Clinical Chemistry, University Hospital Bern, Bern, Switzerland; 3 Division of Human Genetics, University Hospital Bern, Bern, Switzerland; 4 Institute of Physiology, University of Zurich, Zurich, Switzerland; 5 Zurich Center for Integrative Human Physiology (ZIHP), Zurich, Switzerland; 6 Laboratory for Muscle Plasticity, Balgrist University Hospital, Department of Orthopaedics, University of Zurich, Zurich, Switzerland; 7 Institute of Human Movement Sciences, ETH Zurich, Zurich, Switzerland

**Keywords:** Mitochondrial respiration, Citrate synthase, Mitochondrial respiratory chain, Neuromuscular disease

## Abstract

**Background:**

Mitochondrial dysfunction may represent a pathogenic factor in Huntington disease (HD). Physical exercise leads to enhanced mitochondrial function in healthy participants. However, data on effects of physical exercise on HD skeletal muscle remains scarce. We aimed at investigating adaptations of the skeletal muscle mitochondria to endurance training in HD patients.

**Methods:**

Thirteen HD patients and 11 healthy controls completed 26 weeks of endurance training. Before and after the training phase muscle biopsies were obtained from *M. vastus lateralis*. Mitochondrial respiratory chain complex activities, mitochondrial respiratory capacity, capillarization, and muscle fiber type distribution were determined from muscle samples.

**Results:**

Citrate synthase activity increased during the training intervention in the whole cohort (*P* = 0.006). There was no group x time interaction for citrate synthase activity during the training intervention (*P* = 0.522). Complex III (*P* = 0.008), Complex V (*P* = 0.043), and succinate cytochrome c reductase (*P* = 0.008) activities increased in HD patients and controls by endurance training. An increase in mass-specific mitochondrial respiratory capacity was present in HD patients during the endurance training intervention. Overall capillary-to-fiber ratio increased in HD patients by 8.4% and in healthy controls by 6.4% during the endurance training intervention.

**Conclusions:**

Skeletal muscle mitochondria of HD patients are equally responsive to an endurance-training stimulus as in healthy controls. Endurance training is a safe and feasible option to enhance indices of energy metabolism in skeletal muscle of HD patients and may represent a potential therapeutic approach to delay the onset and/or progression of muscular dysfunction.

**Trial registration:**

ClinicalTrials.gov NCT01879267. Registered May 24, 2012.

## Background

Huntington disease (HD) is a hereditary, relentlessly progressive neurodegenerative disorder caused by a CAG triplet repeat expansion of the *HTT* gene encoding the protein huntingtin. The disease is characterized by a variety of symptoms, including motor, cognitive and psychiatric dysfunctions [1]. Huntingtin is found in most tissues, encompassing the central nervous system and peripheral tissue, including skeletal muscle. So far, most research has focused on the role of huntingtin in central nervous tissue. Hence, its role and according pathomechanisms in peripheral tissue, as in skeletal muscle, remains scarce. Previous studies reported mitochondrial abnormalities in skeletal muscle of HD patients [2, 3] and diminished skeletal muscle mitochondrial oxidative metabolism [4, 5]. In a previous study, we showed that maximal oxidative phosphorylation capacity and mitochondrial respiratory capacity of complex I are reduced in HD patients compared to healthy controls [6]. Collectively, these studies assume that mitochondrial dysfunction may represent a pathogenic factor in HD.

In a HD mouse model, physical activity attenuates the onset of specific motor symptoms [7], improves coordination and gait function and retards disease progression [8]. The underlying reasons for these improvements remain inconclusive, especially as predominantly neuronal and cognitive mechanisms have been investigated [7, 8]. Thus, evidence for a positive effect of physical activity on mitochondrial function in HD skeletal muscle is lacking both in humans and animal models. In a recent publication, we showed that endurance exercise increased peak oxygen uptake (*V̇*O_2peak_) to a similar extent in HD patients and healthy controls [9]. In addition, in healthy humans, endurance training is well known to improve mitochondrial properties. Hence, endurance training could potentially enhance physical performance as well as oxidative capacity in HD patients.

In the present study, we aimed at investigating adaptations of the skeletal muscle and mitochondria to endurance training in HD patients. Based on our previous results [6], we hypothesized that skeletal muscle mitochondrial function is impaired in HD patients, but that it can be increased by endurance exercise to a similar extent as in healthy controls.

## Methods

### Participants

Fifteen male HD patients and fifteen age-matched male controls were included in the study. One HD patient and three control participants prematurely finished the study due to loss of motivation. The data of one HD patient were excluded from the analysis because he was diagnosed with leukemia during the training intervention. In one control participant, the amount of tissue obtained from biopsy was not sufficient for all analyses. Finally, thirteen HD patients with a genetically verified diagnosis of HD (age: 53.2 ± 8.8 years, height: 175 ± 5 cm, body mass: 78.7 ± 13.0 kg, *V̇*O_2peak_: 36.1 ± 6.5 ml·min^−1^·kg^−1^, CAG repeats: 42.0 ± 2.3) and eleven control participants (age: 49.7 ± 6.8 years, height: 178 ± 4 cm, body mass: 84.6 ± 13.6 kg, *V̇*O_2peak_: 36.5 ± 6.0 ml·min^−1^·kg^−1^) were included in the analyses. There were no statistically significant differences between groups for age, height, body mass, and *V̇*O_2peak_. In the Unified Huntington Disease Rating Scale, HD patients had the following scores: total motor score 18.0 ± 8.7, verbal fluency 27.8 ± 10.7, symbol digit modalities 39.3 ± 8.7, Stroop test color 54.1 ± 15.5, Stroop test word 79.6 ± 16.7, Stroop test interference 34.9 ± 11.2, total behaviour score 10.3 ± 12.0, functional checklist score 23.1 ± 2.3, independence scale 91.5 ± 8.5%, total functional capacity 11.0 ± 2.2. These values are compatible with a mostly low disease severity. After completing a routine health questionnaire, the participants were informed about the applied procedures and the associated risks. Participants provided written informed consent for participation in this study. All experiments were approved by the local ethics committee and the study was performed in accordance with the ethical standards laid down in the Declarations of Helsinki for human experimentation. Medication was protocolled during the entire study period. None of the HD patients took drugs that enhance muscle mitochondrial function.

### Experimental design

The natural course of the disease was observed in HD patients for 26 weeks, before endurance training started. Thereafter, HD patients and healthy controls conducted an endurance training intervention for a total of 26 weeks. In HD patients, before the observation phase of the natural disease progression, a baseline percutaneous muscle biopsy was obtained from *M. vastus lateralis*. A second biopsy was obtained from HD patients before the training intervention and a final third biopsy directly after the training intervention. In healthy controls, only two biopsies (before and after the training intervention) were obtained. Muscle samples were used for histochemistry, mitochondrial DNA quantification, spectrophotometric analyses of the respiratory transport chain and mitochondrial respiration measurements.

### Exercise intervention

The exercise intervention was divided into three training phases that were interspersed with 1-week rest periods. The rest periods served its role in supporting (psychological) recovery from the previous demanding training phases. During the first 10 weeks, HD patients and healthy controls followed a constant-load cycling protocol (30 min at a cycling power corresponding to 65% *V̇*O_2peak_) three times a week. After the first rest period, HD patients and healthy controls performed a high-intensity interval training (4 × 4 min at 90–95% of peak heart rate with 3 min low-intensity rest intervals at 70% of peak heart rate, according to Helgerud et al. [10]) 3 times a week for the following 8 weeks. After the second 1-week rest period, a final 6-weeks endurance training protocol consisting of two interval training sessions and one constant-load training session per week [11] was performed. To assure a sufficient training stimulus, cycling power was increased as soon as the participants’ heart rate fell below the target values.

### Skeletal muscle biopsy

Before each biopsy, participants were tested for coagulation abnormalities (Quick/INR, number of thrombocytes). Skeletal muscle biopsies were obtained under standardized conditions from the *M. vastus lateralis* upon local anesthesia with 1% lidocaine of the skin and superficial muscle fascia, using the Bergström technique with a needle modified for suction [12]. The biopsy was immediately dissected macroscopically free of fat and connective tissue and divided into sections for actual measurement of mitochondrial respiratory capacity, mitochondrial DNA quantification, spectrophotometrical analysis and histochemistry. Skeletal muscle biopsies were conducted before and after the training intervention. In HD patients, an additional biopsy was obtained at baseline.

### Mitochondrial respiration measurements

In a subgroup of six HD patients, mitochondrial respiration measurements were performed before and after the training intervention. The preparation of the skeletal muscle tissue and the titration protocol were conducted as described in detail previously [13]. In summary, after mechanical muscle fiber separation, chemical permeabilization in biopsy preservation solution and washing in mitochondrial respiration medium 05, muscle bundles were blotted dry and measured for wet weight (ww) on a balance-controlled scale (XS205 DualRange Analytical Balance, Mettler-Toledo AG, Greifensee, Switzerland). Respiration measurements were subsequently performed in mitochondrial respiration medium 06. Oxygen consumption of the individual muscle tissue was thereby measured at 37 °C using the high-resolution Oxygraph-2 k (Oroboros, Innsbruck, Austria). Experiments were performed as duplicates in a hyperoxygenated environment in order to prevent any potential oxygen diffusion limitation. Thereby, oxygen concentration ranged between 200 and 450 nmol mL^−1^ within the chambers. The respiratory measurement protocol was specific to the analysis of individual aspects of respiratory capacity and coupling control efficiency during several substrate states induced via separate titrations. All titrations were added in series as presented, whereby the concentrations of substrates, uncouplers and inhibitors used were based on prior experiments [14]. The titration protocol was modified from previous protocols where they are described in detail [14].

### Biochemical assays

Skeletal muscle homogenates were performed as described previously [15, 16]. The activities of the individual respiratory chain complexes and the mitochondrial matrix marker enzyme citrate synthase were measured spectrophotometrically with an UV-1601 spectrophotometer (Shimadzu, Kyoto, Japan) using 1 ml sample cuvettes thermostatically maintained at 30 °C according to Birch-Machin and Turnbull [17]. Values were estimated by the difference in activity levels measured in the presence and absence of specific inhibitors and expressed as ratios to the mitochondrial marker enzyme citrate synthase (mU·mU^−1^ citrate synthase), which was determined as described [18].

### Histochemistry

Tissue sections were cut at 12-μm thickness in a cryostat maintained at **−**25 °C, and mounted on Fisherbrand Superfrost/Plus microscope slides (Fisher Scientific, Pittsburgh, PA, USA). The serial cryocut cross-sections were stained using the myofibrillar adenosine triphosphatase (mATPase) method at pH 4.6 as previously described [19]. Muscle fibers were classified according to their myosin heavy chain (MyHC) isoform into MyHC-1, MyHC-2A and MyHC-2X. Per point in time, 479 ± 300 MyHC-1 fibers, 257 ± 136 MyHC-2A fibers, and 94 ± 109 MyHC-2X fibers were counted for determination of muscle fiber type distribution. As marker for muscle capillaries, the monoclonal mouse anti-human CD31 endothelial antibody (DAKO, Carpinteria, CA, USA, 1:600 dilution) was used. Overall capillary-to-fiber ratio was calculated by dividing the number of CD31-positive structures by the number of muscle fibers. On average, 159 ± 96 capillaries were counted per point in time for the calculation of the overall capillary-to-fiber ratio. We used the NIH Image J Software (version 1.44o, National Institutes of Health, Bethesda, MD, USA) for all fiber analyses.

### Statistical analysis

Data are presented as mean ± SD. Normality of data was visually ascertained by Q-Q-Plots. Analysis of variance with repeated measures was performed to detect changes between groups over time during the intervention phase. To detect differences between baseline and pre-training in the HD group, paired samples *t*-tests were performed. SPSS statistical software (Version 24.0, SPSS, Chicago, IL, USA) was used for all statistical analyses. A value of *P* < 0.05 was set as statistical significance.

## Results

Citrate synthase activity increased in the HD group by 18.9% and in the control group by 27.5% during the training intervention (Table 1). There was a group effect for citrate synthase activity, but no group x time interaction was present during the training phase (*P* = 0.522). Absolute complex I activity decreased during the natural observation period in HD patients by −15.6%. There was no further decrease in absolute complex I activity during the training intervention in HD patients (−0.7%). Healthy controls had a significantly higher absolute complex I activity as compared to HD patients. There were time effects for absolute complex III, complex V and succinate cytochrome c reductase (SCCR) activities during the training intervention. Neither group nor group x time effects were present for these variables. Complex I activity normalized to citrate synthase activity decreased during the natural course observation phase in HD patients by −15.6%. Moreover, there were significant time effects for relative complex I (−16.2% and −22.5% for the HD and control group, respectively) and complex II (−13.6% and −1.3% for the HD and control group, respectively) activities during the training intervention. HD patients exhibited significant higher relative complex V and NADH cytochrome c reductase (NCCR) activities than healthy controls. There were no group x time interactions for all assessed relative variables (Table 1).

**Table 1:** Mitochondrial complex activities at baseline, pre-training and post-training in Huntington disease (HD) patients and healthy controls

	HD (*n* = 13)			Controls (*n* = 11)	
	Baseline	Pre-Training	Post-Training	Pre-Training	Post-Training
*Absolute values*					
Citrate synthase (mU·mU^−1^ Protein)	150 ± 42	141 ± 34	163 ± 47^##, †^	164 ± 65	198 ± 67^##, †^
Complex I (mU·mU^−1^ Protein)	35 ± 11	28 ± 9*	26 ± 7^†^	36 ± 15	33 ± 13^†^
Complex II (mU·mU^−1^ Protein)	40 ± 9	38 ± 11	37 ± 11	37 ± 14	44 ± 16
Complex III (mU·mU^−1^ Protein)	123 ± 35	114 ± 32	137 ± 51^##^	122 ± 46	154 ± 55^##^
Complex IV (mU·mU^−1^ Protein)	165 ± 39	151 ± 56	131 ± 47	142 ± 50	164 ± 51
Complex V (mU·mU^−1^ Protein)	75 ± 25	72 ± 24	91 ± 29^#^	69 ± 29	80 ± 26^#^
SCCR (mU·mU^−1^ Protein)	34 ± 11	35 ± 8	49 ± 25^##^	35 ± 14	46 ± 14^##^
NCCR (mU·mU^−1^ Protein)	28 ± 15	29 ± 12	35 ± 13	26 ± 11	30 ± 10
*Relative values*					
Complex I (mU·mU^−1^ CS)	0.241 ± 0.044	0.201 ± 0.051*	0.164 ± 0.037^###^	0.225 ± 0.049	0.168 ± 0.044^###^
Complex II (mU·mU^−1^ CS)	0.271 ± 0.041	0.269 ± 0.059	0.227 ± 0.038^#^	0.232 ± 0.044	0.225 ± 0.043^#^
Complex III (mU·mU^−1^ CS)	0.837 ± 0.158	0.828 ± 0.203	0.846 ± 0.184	0.796 ± 0.087	0.779 ± 0.099
Complex IV (mU·mU^−1^ CS)	1.124 ± 0.191	1.067 ± 0.300	0.835 ± 0.246	0.871 ± 0.119	0.840 ± 0.133
Complex V (mU·mU^−1^ CS)	0.507 ± 0.155	0.522 ± 0.168	0.528 ± 0.109^†^	0.414 ± 0.074	0.400 ± 0.067^†^
SCCR (mU·mU^−1^ CS)	0.228 ± 0.056	0.253 ± 0.048	0.269 ± 0.095	0.215 ± 0.033	0.236 ± 0.034
NCCR (mU·mU^−1^ CS)	0.185 ± 0.094	0.209 ± 0.081	0.222 ± 0.120^††^	0.145 ± 0.029	0.153 ± 0.016^††^

Values are mean ± SD. NCCR, NADH cytochrome c reductase, SCCR, succinate cytochrome c reductase. *P < 0.05, significant different to baseline; ^#^ *P* < 0.05, ^##^ *P* < 0.01, ^###^ *P* < 0.001, significant time effect; ^†^ *P* < 0.05, ^†^ *P* < 0.01, significant group effect

**Fig. 1:**
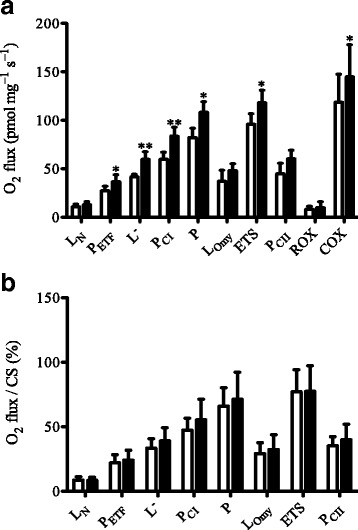
Mass-specific mitochondrial (**a**) and mitochondrial-specific (**b**) respiratory capacity (normalized to citrate synthase). Respiratory capacities are presented for Huntington disease patients (*n* = 6) before training (white bars) and after the training intervention (black bars). L_N_, leak respiration without adenylates; P_ETF_, fatty acid oxidative capacity; P_CI_, respiratory capacity of complex I; P, oxidative phosphorylation capacity; L_Omy_, oligomycin-induced leak respiration; ETS, electron transport system capacity; P_CII_, respiratory capacity of complex II; ROX, residual oxygen consumption; COX, respiratory capacity of complex IV. Values are mean ± SD. *P < 0.05; **P < 0.01, significant differences between pre-training and post-training

In the subgroup of six HD patients with mitochondrial respiratory measurements, fatty acid oxidative capacity (+38.7%), leak respiration without adenylates (+23.6%), respiratory capacity of complex I (+43.2%), oxidative phosphorylation capacity (+33.4%), maximum electron transport chain capacity (+24.6%) and respiratory capacity of complex IV (+23.4%) were increased after the training intervention (Fig. 1a). After normalizing mitochondrial respiratory capacity to citrate synthase activity, all respiratory states remained unaltered in response to endurance training (Fig. 1b).

**Fig. 2:**
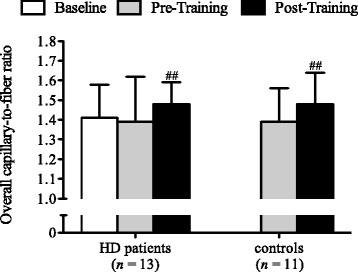
Overall capillary-to-fiber ratio in Huntington disease patients (HD) and healthy controls at baseline (white bars), pre-training (grey bars) and post-training (black bars). ^##^ *P* < 0.01, significant time effect

Muscle fiber type distribution remained constant during the endurance training intervention in both groups (Table 2). Additionally, there was no group effect for muscle fiber type distribution. There was also no alteration in muscle fiber type distribution in the HD group during the natural course observation period. Overall capillary-to-fiber ratio remained constant during the natural course observation period in HD patients (*P* = 0.449). During the endurance training intervention, overall capillary-to-fiber ratio increased by 8.4% in HD patients and by 6.4% in control participants (Fig. 2). There was no group effect (*P* = 0.973) nor a group x time interaction (*P* = 0.928) for the overall capillary-to-fiber ratio.

**Table 2:** Muscle fiber type distribution and cross-sectional areas of muscle fibers at baseline, pre-training and post-training in Huntington disease (HD) patients and healthy controls

	HD patients (*n* = 13)			Controls (*n* = 11)	
	Baseline	Pre-Training	Post-Training	Pre-Training	Post-Training
*Muscle fiber type distribution*					
MyHC-1 (%)	59.0 ± 13.9	57.0 ± 13.8	60.2 ± 10.0	56.7 ± 9.7	53.5 ± 12.2
MyHC-2A (%)	33.3 ± 9.5	30.9 ± 10.6	32.5 ± 10.7	31.7 ± 7.5	34.9 ± 12.9
MyHC-2X (%)	7.8 ± 6.7	12.1 ± 10.0	7.3 ± 5.8	11.6 ± 10.0	11.6 ± 7.3

Values are mean ± SD. MyHC, myosin heavy chain

## Discussion

In this study, we showed that increases in mitochondrial content and capillarization in response to prolonged endurance training is similar for HD patients and matched healthy controls. Namely, after an endurance training intervention, citrate synthase, complex III, complex V and SCCR activities increased similarly in HD patients and healthy controls. In addition, an increase in mass-specific mitochondrial respiratory capacity was present in HD patients during the endurance training intervention. Furthermore, overall capillary-to-fiber ratio increased in HD patients and healthy controls following endurance training.

The most important aspect of the present study is the similar responsiveness of the skeletal muscle of HD patients to an endurance training stimulus when compared to healthy controls. Evidence for this is that citrate synthase activity significantly increased in both training groups. Moreover, several mitochondrial complex activities increased (in absolute values), which is in line with the increased mass-specific respiratory capacities of the mitochondrial complexes. Altogether, these results implicate that mitochondrial content was increased in response to endurance training in HD patients as well as in healthy controls. Increased citrate synthase activity is a well-known adaptation to moderate-intensity continuous training and/or high-intensity interval training in healthy participants and patients within a wide variety of diseases [20]. In addition, overall capillary-to-fiber ratio increased similarly in HD patients and controls. These findings are consistent with our previous result showing that *V̇*O_2peak_ is increased in HD patients following a period of endurance training [9].

Our results emphasize that increasing mitochondrial biogenesis is possible in HD patients in response to endurance training. Since post-training values of HD patients for citrate synthase activity approached pre-training values of healthy controls, it might be that life-long endurance training in HD patients could have the potential to delay mitochondrial dysfunction in skeletal muscle. The present results expand the findings of pharmacological studies in the HD mouse model aimed at delaying mitochondrial alterations [21–23]. For instance, the pan-peroxisome proliferator-activated receptor agonist bezafibrate prevented structural abnormalities of mitochondria in HD mice [21, 22]. Moreover, it is postulated that increasing mitochondrial biogenesis does not only prevent mitochondrial dysfunction but also attenuates neurodegeneration and disease progression [24]. Therefore, an elevation of mitochondrial biogenesis through endurance training might be a promising therapeutic intervention in HD patients.

When values for mitochondrial enzyme activities and mitochondrial respiratory capacities were normalized for citrate synthase activities, no significant increases were observed.

These results indicate that an increase in mitochondrial content or quantity was present, but that intrinsic mitochondrial function or quality remained constant. Our finding is supported by a previous study investigating the effects of moderate intensity continuous exercise or high-intensity interval training in healthy participants [25]. Based on these observations, it has recently been postulated that mitochondrial quality improvements do only occur once mitochondrial content has increased to the limit allowed by spatial constraints of the muscle fibers [26].

HD patients exhibited lower citrate synthase and absolute complex I activity when compared to their healthy counterparts. The lower citrate synthase activity in conjunction with similar relative enzyme activities indicated that HD patients exhibited a lower mitochondrial content but preserved mitochondrial quality when compared to healthy controls. This finding is in line with a previous finding of our group [6] and expands the knowledge about mitochondrial dysfunction in HD patients. Up to date, it was reported that mitochondrial abnormalities are present in skeletal muscle of HD patients [2, 3] and that some patients had diminished skeletal muscle mitochondrial oxidative metabolism [4, 5].

Absolute and relative complex I activities decreased during the natural course observation phase in HD patients. Moreover, relative complex I and II activities decreased during the training intervention in the entire cohort. This observation might rely on two different factors. First, respiratory states of complex I and II normalized for citrate synthase activity were not altered in the subgroup of HD patients. Therefore, the discrepancy in relative complex I and II activities measured by spectrophotometry or mitochondrial respiration might be based on methodological differences. The spectrophotometric measurements reflect isolated respiratory chain complex activities from muscle homogenates, while mitochondrial respiration measurements in permeabilized muscle fibers measures oxygen consumption dependent on different substrates. The latter procedure preserves mitochondrial morphology and integrity, whereby the examination of the intact mitochondrial network is feasible [27]. Therefore, mitochondrial respiration measurements might be considered as more accurate measurements to assess in vivo mitochondrial function. Second, associations between the degree of the disease and complex I and II/III dysfunctions, assessed spectrophotometrically, have been reported previously [2, 28]. Hence, it might be assumed that the HD patients of this study, which exhibited mostly low disease severity, were only slightly affected by these dysfunctions. Prolonged disease duration and a higher disease severity might, however, lead to lower complex activities and reduced mitochondrial quality.

In contrast, complex V and NCCR activities normalized to citrate synthase activity were higher in HD patients as compared to healthy controls. We can only speculate about the underlying reasons for these elevated activities. The elevated complex V activity might rely on an increased substrate availability. Choreatic movements in HD patients might elevate intracellular adenosine diphosphate (ADP) concentrations, whereby a higher amount of substrate is available for adenosine triphosphate (ATP) synthase. In this regard, ADP availability would be the rate-limiting step in healthy individuals and would explain their lower complex V activity. In HD patients, the higher relative NCCR activity and the tendency for a higher relative SCCR activity indicate that complex I and II are, at the current disease state, not the rate-limiting steps in the respiratory transport chain. Moreover, the (unknown) rate-limiting step between complex I or II and complex III is even improved in HD patients compared to healthy controls. This improvement in the rate-limiting step might represent an early onset compensation for the disease-related dysfunctions in complex I and II/III activities, as previously reported [2, 28].

## Conclusions

In conclusion, skeletal muscle mitochondria of HD patients are equally responsive to a prolonged period of endurance training as healthy controls. Moreover, besides mitochondrial content, overall capillary-to-fiber ratio increased similarly in HD patients and healthy controls. In contrast, there were no alterations in muscle fiber type distribution in both groups. Therefore, endurance training is a safe and feasible option to enhance energy metabolism in skeletal muscle of HD patients and may represent a potential therapeutic approach to delay the onset or progression of muscular dysfunction.
